# Multifrequency-based sharpening of focal volume

**DOI:** 10.1038/s41598-022-25886-9

**Published:** 2022-12-21

**Authors:** Thomas Riis, Jan Kubanek

**Affiliations:** Department of Biomedical Engineering, University of Utah, Salt Lake City, 84102 USA

**Keywords:** Applied physics, Translational research

## Abstract

Systems that emit electromagnetic or sonic waves for diagnostic or interventional applications often have constraints on the size of their aperture, and thus produce an elongated focus in the axial dimension. This extended depth of focus limits imaging resolution and spatial specificity of the delivered energy. Here, we have developed a method that substantially minimizes the depth of focus. The method superimposes beams of distinct frequencies in space and time to create constructive interference at target and amplify deconstructive interference everywhere else, thus sharpening the focus. The method does not require labeling of targets or other manipulations of the medium. Using simulations, we found that the method tightens the depth of focus even for systems with a narrow bandwidth. Moreover, we implemented the method in ultrasonic hardware and found that a 46.1% frequency fractional bandwidth provides an average 7.4-fold reduction in the focal volume of the resulting beams. This method can be readily applied to sharpen the focus of interventional systems and is expected to also improve the axial resolution of existing imaging systems.

## Introduction

Systems that emit electromagnetic or sonic waves have powered critical diagnostic and interventional applications^1–3^. Generally in these applications, there is a need to minimize focal size to increase imaging resolution and spatial specificity. Reducing the focal size can be achieved by increasing the emitting aperture dimensions^4,5^. However, a defining feature of many of these systems, including radar and ultrasonic transducers, is that their aperture dimensions are limited by spatial or hardware constraints. In particular, the diameter *D* of the transmitting aperture is often relatively small with respect to the distance *f* within which the system operates. The limited aperture size has led to a fundamental problem: an elongated depth of focus^6–10^. This problem is severe because the depth of focus is proportional to $\left( \frac{f}{D}\right) ^2$^7,11^. Therefore, a reduction in the aperture size leads to a squared increase in the length of the focal region.

**Figure 1:**
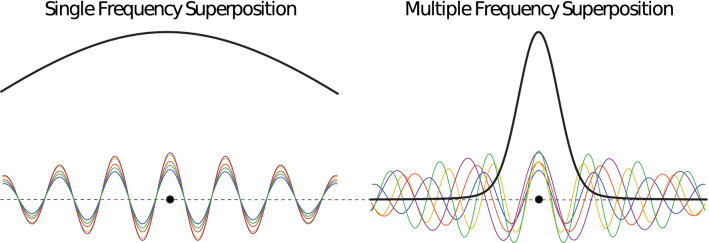
Label-free sharpening of focal field by emitting waves at distinct frequencies and at controlled times. In traditional emission beams (left), a superposition of waves of a single frequency leads to an elongated beam (solid black line). MFS (right) uses multiple frequencies emitted at times such as to amplify destructive interference outside the target, thus sharpening focus. The target is represented by the black dot.

The resulting elongated, cigar-shaped focal regions have limited both imaging and interventional applications. Optical imaging systems have overcome this issue by using opposing objective methods with increased aperture size^12–14^ or methods that label or otherwise alter the imaged target or region^15–19^. Ultrasound imaging systems have also improved spatial resolution through target labeling^20,21^ in addition to increasing aperture size^22,23^ to decrease focal volume. However, increasing a system’s aperture or labeling the targets can be impractical or impossible, especially in domains other than imaging. Interventional or therapeutic applications require a minimization of the focal volume to specifically manipulate the desired target while sparing surrounding regions. For example, ultrasound ablative surgeries of the brain use an array of transducers with aperture size constrained to a hemisphere that surrounds the skull calvaria^24^. The array’s focal size determines the minimum volume of the brain that can be ablated and thus must be minimized to avoid destroying healthy tissue surrounding the target. In interventional applications in general, focal size has been minimized by employing increased apertures^4,5,25,26^, and wave based methods such as increasing center frequency^27^and modulating a carrier wave with another offset or low frequency wave^26,27^. However, as stated above, practical limits exist in most applications on both increasing the aperture size and increasing center frequency. Since high frequency waves are more easily absorbed and attenuated, the use of low frequencies is often the only choice when targeting deep areas of the body.

A method to sharpen the focus without increasing the aperture size, labeling the medium, or increasing the center frequency would provide a practical solution to improving imaging resolution and spatial selectivity in imaging and therapeutic wave emitting systems. To address this fundamental issue, we have developed a method that substantially sharpens the depth of focus for limited apertures. The method is related to opposing objective methods in that it uses two opposing apertures but critically, it does not require an increase in aperture size. Instead, the method tightens the focal region by superimposing a range of frequencies in space and time to ensure constructive interference at target and enhance deconstructive interference outside the target (Fig. 1). This multifrequency superposition is henceforth referred to as MFS. MFS is designed to be practical and applicable to systems with limited bandwidth. We implemented MFS in hardware and confirmed a substantial reduction of the focal volume in an ultrasonic system with limited bandwidth. We simulated the same hardware system and found an exponentially decreasing relationship between focal volume and fractional bandwidth. Further, we found the reduction in focal volume was superior to focusing with only the highest frequency in the superposition.

## Results

**Figure 2:**
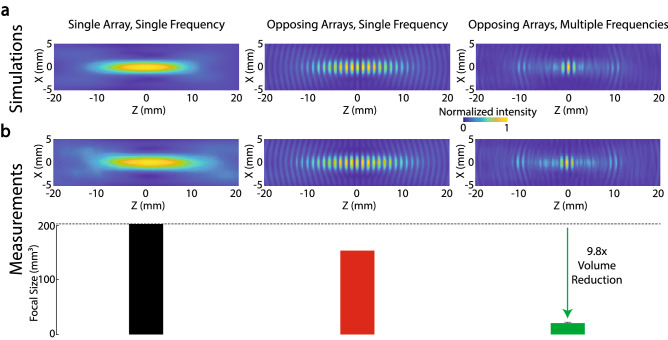
MFS performance. Simulated (**a**) and measured (**b**) fields provided by the traditional single-frequency emission from a single aperture (left column), single-frequency emission from opposing apertures (middle column), and MFS (right column). The corresponding focal volumes are quantified using the bars on the bottom (mean ± s.d.).

We validated the MFS concept using simulations that compare the fields produced by MFS with those of traditional, single-frequency approaches. All cases used spherically focused phased arrays of 126 elements (Methods). In the first case, we emitted a single frequency (650 kHz) from a single array. Figure 2a, left shows that this traditional approach produces a characteristic elongated beam. The beam had a focal volume of 112.92 mm$^3$. Next, we tested the effect of opposing apertures^12–14^. The opposing apertures produced the expected standing wave pattern^28^, and reduced the focal volume by a modest 5.3%, to 106.89 mm$^3$ (Fig. 2a, middle). Critically, applying MFS to the same opposing array geometry reduced the focal volume by 86.0% (Fig. 2a, right), to an average 15.82 ± 0.51 mm$^3$ (mean ± s.d.). Thus, MFS provided a 6.76 ± 0.21 (mean ± s.d.) reduction of the focal volume compared with the same geometry not using MFS, and this difference was significant ($t_{19} = 800.5$, $p=1.74\times 10^{-44}$, two-tailed t-test).

We next implemented these simulations in ultrasonic hardware and measured the pressure fields using a hydrophone (see Methods). The resulting fields are shown in Fig. 2b, in the same format as in Fig. 2a. The single array and opposing arrays driven at the single frequency had focal volumes of 203.37 mm$^3$ and 154.47 mm$^3$, respectively. Critically, in line with the simulations, MFS reduced the focal volume substantially, to a mere 20.75 ± 1.79 mm$^3$ (mean ± s.d.). Thus, compared to the opposing arrays at center frequency, MFS provided a 7.44 ± 0.59 (mean ± s.d.) reduction of the focal volume, and this difference was significant ($t_{19} = 298.09$, $p=1.03\times 10^{-29}$, two-tailed t-test).

**Figure 3:**
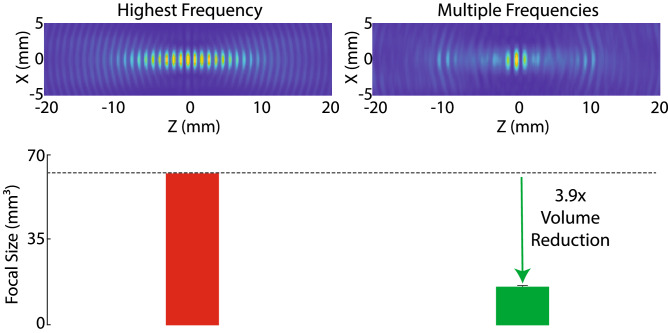
MFS generates sharper focus than the highest available frequency alone. Left: The field produced by the highest frequency available within the MFS bandwidth. Right: MFS. Error bars represent the s.d. Both fields were obtained using simulations analogous to those of Fig. 2a.

MFS used a 46% fractional bandwidth (500–800 kHz) with respect to the central frequency (650 kHz) used by the single-frequency approaches. We tested that the improvements in the focal volume were not simply due to the presence of higher frequencies (i.e., frequencies over 650 kHz) in the bandwidth. Fig. 3 shows that this is not the case. MFS (right) reduced the focal volume substantially also with respect to the single-frequency approach operating at the highest available frequency (800 kHz; left). Specifically, the volume reduced from 106.89$^3$ to 15.82 ± 0.50 mm$^3$ (mean ± s.d.), i.e., by a factor of 3.9 ± 1.3, and the difference was significant ($t_{19} = 408.77$, $p=6.12\times 10^{-39}$, two-tailed t-test).

**Figure 4:**
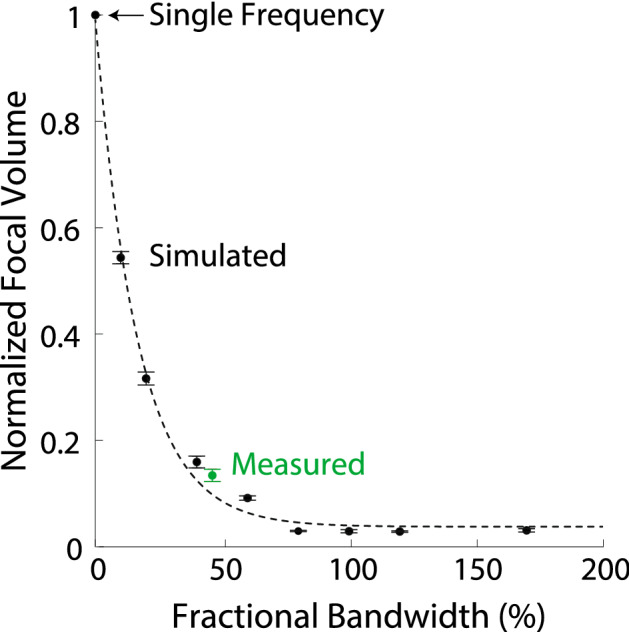
MFS gains as a function of available bandwidth. Mean ± s.d. focal volume as a function of fractional bandwidth, relative to the single frequency case (0% bandwidth). The data are provided separately for simulations for each datapoint (black; $n=7$), and the measurement of Fig. 2b (green). The black dotted line represents an exponential fit to the data ($f(x) = e^{-0.06\ x}$).

Next, we tested how the MFS effect depends on the available bandwidth. Figure 4 shows the focal volumes for fractional bandwidths in the range from 0 to 170%, using simulations (black) and the measurement of the output of our hardware implementation (green). We found that the focal volume decreases exponentially with the available bandwidth (98% of variance explained in the data points). This exponential effect results in large reductions in focal volume even for systems with limited bandwidth. For instance, a fractional bandwidth of 10% yielded a 1.85 ± 0.04 (mean ± s.d.) reduction of the original single frequency focal volume (0% bandwidth). An 80% bandwidth leads to 33.68 ± 1.4 (mean ± s.d.) factor reduction in the focal volume compared to the single-frequency case (2.9 ± 0.1% of single frequency focal volume).

**Figure 5:**
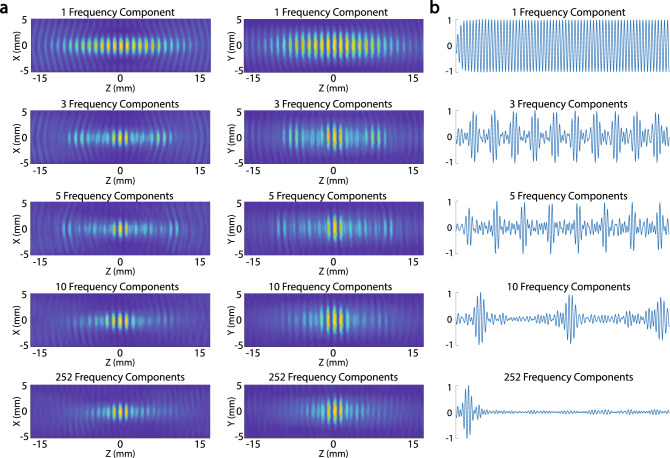
Fields and waveforms at target for all measured frequency combinations. (**a**) Same hardware and approach as in Fig. 2b, but now separately (rows) for 1, 3, 5, 10, and 252 frequency components (equally spaced between 500 and 800 kHz) and separately for the X (left column) and Y (middle column) components of the fields. (**b**) The waveforms at target that result from the superposition of the particular number of frequencies.

Finally, we tested how the MFS effect depends on specific selections of the frequency distribution within the available bandwidth (Fig. 5). There was a trend toward more frequency values providing a sharper focus (Fig. 5a), but there was local deviation from this observation e.g., 5 equally distributed frequencies (Fig. 5a). This complex issue deserves a systematic investigation in future studies. As expected, the superposition of frequencies could produce complex waveform at the target (Fig. 5b). The more frequency components available, the more impulse-like the waveform at the target, as expected from the Fourier theory. These observations are based on continuous waveforms; which signals can be produced by pulsed waveforms should also be investigated in future studies.

## Discussion

We devised a label-free method, MFS, that substantially improves the depth of focus of wave-based radiation beams. MFS rests on a controlled superposition of waves and does not require labeling or a modification of elements within the target space. We implemented the method in standard ultrasonic hardware and validated that the depth of focus can be reduced substantially even for systems with relatively narrow bandwidth.

MFS is based on a timed emission of waves to achieve constructive interference at the target of interest. Critically, even a small variation in the frequencies emitted from the individual transducers is sufficient to amplify destructive interference near the target, thus leading to substantial sharpening of the depth of focus (Figs. 1 and 2). The multifrequency emission is necessary for this effect; using the highest frequency within the bandwidth alone produces a much larger focus (Fig. 3).

The multifrequency nature of MFS distinguishes it from previous label-free methods. Nonetheless, MFS incorporates an important concept that has been harnessed in optics on several occasions. Specifically, MFS uses two apertures that oppose each other, akin to opposing objective methods in optical imaging^12–14^. However, unlike in optics, MFS does not require an increase in the aperture or the solid angle to improve the depth of focus. The improvement is achieved for a fixed, limited aperture by emitting waves of multiple frequencies at defined times to achieve constructive interference at the target while amplifying destructive interference elsewhere. For single frequencies, this geometry produces standing waves (Fig. 2). In optics, this effect alone has been harnessed for improving the axial resolution for imaging purposes^28^. MFS goes beyond this step, applying multifrequency superposition to sharpen the focal volume. This way, MFS is also applicable for interventional or therapeutic applications for which the standing-wave pattern itself would not present a notable or desirable property (Fig. 2).

Label-free improvement in spatial focus can also be achieved using superoscillation. Superoscillation applies complex, optimized lenses to focus waves into focal regions whose size evades the Rayleigh criterion^29^. However, the focal benefit comes at the cost of efficiency—the main lobe receives only a few percent of the total energy, while a large portion of energy is dissipated in side lobes^29,30^. Therefore, although the concept of superoscillation may prove useful for imaging applications, it is unlikely to serve a major role in therapeutic applications. Compared with superoscillation, in MFS, side lobes are smaller than the main lobe (Fig. 2), so the method does not suffer from this issue and is therefore suited also for interventional applications. Furthermore, no lenses are required.

Several previous studies, in particular within ultrasonics, have used multiple frequencies to improve spatial resolution^31–35^. However, these methods, including frequency compounding in elasticity imaging, apply or receive the individual frequency components in separation. The improvement in spatial resolution follows the standard diffraction-limited resolution, in which sharper focus is obtained using higher frequencies. MFS differs fundamentally from these approaches in that MFS emits the distinct frequency components in a controlled spatiotemporal pattern to achieve a specific superposition pattern at the target.

We anticipate that MFS will be particularly useful for interventional and therapeutic applications, which generally require a circumscribed beam. For example, ultrasonic transducers produce a characteristic, cigar-shaped beam^10,36,37^. When applied for therapeutic purposes such as thermal lesioning or neuromodulation, this beam geometry poses a risk of harm to unintended targets. MFS overcomes this limitation (Figs. 2 and 3) and thus is expected to improve the specificity and safety of such treatments. The improvement in the axial resolution may also prove useful in imaging, further increasing axial resolution of existing methods^12–18,28^. The increase in axial resolution is expected to be useful for applications that rest on opposing emitters in general. For instance, this method may boost manipulation capabilities of acoustic tweezers^38^ or planar linear ion traps^39,40^.

MFS harnesses the available bandwidth of wave-emitting systems. We found that the focus improves exponentially with increased bandwidth (Fig. 4). Therefore, even systems with very limited bandwidth may benefit from MFS.

We implemented MFS using standard ultrasonic hardware. The method requires multiple elements and a system that can generate multiple frequencies with precise timing. This may be a relatively minor issues for sound waves, which propagate through media relatively slowly and so do not pose high demands on the control electronics, but could become a constraint for optical systems and possibly also other systems that operate at or near the speed of light. Nonetheless, phase can also be controlled with special lenses^41^, and so the method should be practically implementable also for systems based on electromagnetic waves.

In summary, we developed a wave-based method to overcome the fundamental issue of elongated beams produced by systems with limited aperture. We have demonstrated that the method can be readily implemented in existing hardware and that the reduction in beam length is substantial. This method is expected to particularly benefit interventional and therapeutic applications based on waves, and may further contribute to the reduction of axial resolution in imaging systems.

## Methods

### Aperture geometries and target

**Figure 6:**
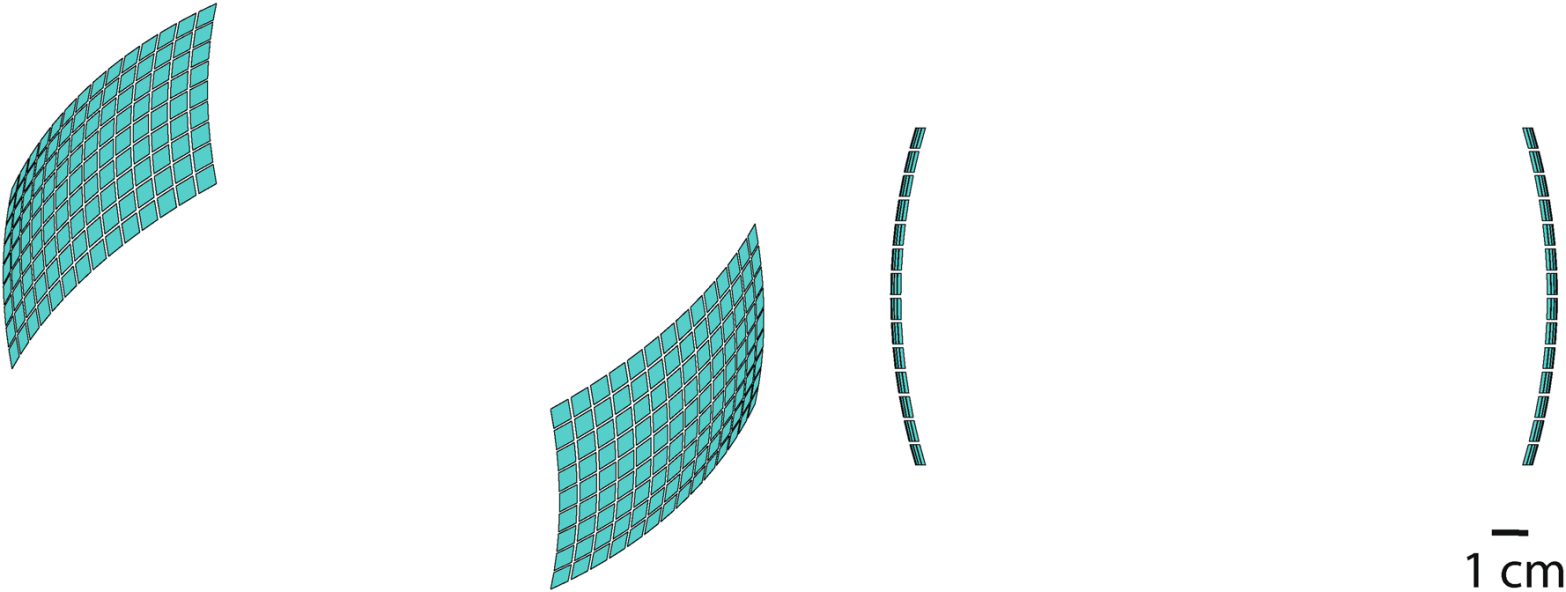
Array geometry. Schematic of the arrays used in the simulations and implemented in hardware. Each square corresponds to an array element.

We used two spherically focused emitter arrays in both simulation and experimental measurements. Each array had a spherical curvature with radius of 165 mm and consisted of 126 individual emitter elements (6 mm $\times$ 6 mm) organized in a 9 $\times$ 14 element grid with inter-element spacing of 0.5 mm. Each array had a height of 55 mm and a width of 86 mm. The beam produced by each array had a geometric focus centered at 85 mm away from the face of the array in the axial dimension. In the analyses that used opposite configurations, the arrays were facing each other, separated by a distance of 170 mm (Fig. 6).

### Ultrasonic hardware

The ultrasonic arrays were made of the PMN-PT material (Doppler Electronic Technologies, Guangzhou, China), and operated at a fundamental frequency of 650 kHz. The individual elements of the arrays were driven by a programmable system (Vantage256, Verasonics, Kirkland, WA).

### MFS

Like traditional beamforming methods, MFS excites multiple elements of a phased array such that the individual waves arrive at a target in phase, at their peak value. However, unlike traditional beamforming, MFS excites each individual element with a distinct frequency that lies within the element’s bandwidth. The delays are set such that the peak of waveform from each element arrives into the defined target simultaneously and thereby constructively interfere. This also leads to amplified destructive interference in the vicinity of the target due to the diminished coherence from multiple frequencies (but not at the target). Frequencies are assigned to each element in the phased array to minimize standing waves with opposing elements.

### Waveform parameters

The available bandwidth can be discretized into an arbitrarily high number of frequencies. We tested five sets of frequencies. In all cases, the frequencies were equally spaced across the transducers’ bandwidth, which ranged from 500 to 800 kHz. We measured the effects of single frequency (650 kHz), three frequencies (500, 650, 800 kHz), five frequencies, ten frequencies, and 252 frequencies (Fig. 5). We found that five frequencies provided a favorable trade-off between sharp focus and the number of necessary frequencies (Fig. 5). Therefore, the simulations and measurements used five frequencies, with the exception of Fig. 4, which used 252 frequencies to fully harness the available bandwidth.

Each element of the array was randomly assigned one frequency from the set. We found that randomizing the frequency assignment across the array geometry minimizes the focal volume. Moreover, assigning the frequencies to the elements randomly allowed us to produce multiple realizations and multiple measurements, which were key for statistical purposes.

Each element was driven at its unique frequency for 153 $\upmu$s, i.e., the duration of 100 cycles at 650 kHz. For the elements of the actual hardware, we made sure to normalize the amplitude output by the frequency characteristic of each element. This way, all frequencies across the 500–800 kHz bandwidth had comparable amplitude.

Ultrasonic transducers require a certain number of cycles to reach maximum amplitude. To take this hardware constraint into account, we delayed the transmission of the waveforms such that their 10th peak arrived at the target at the same time.

### Simulations

The simulations were performed using Field II^42^. The code is available and documented at onetarget.us/download/MFS. We recorded the output over a 10 mm $\times$ 40 mm grid in the XY and XZ planes with 0.15 mm spacing. The waveform at each point in the grid was recorded and saved. Since field amplitudes are additive, the total pressure was computed as the sum of the contributions of the individual elements.

### Measurements

The ultrasonic pressure fields were measured using hydrophone field scans. Specifically, the fields were measured using a capsule hydrophone (HGL-0200, Onda) secured to 3-degree-of-freedom programmable translation system (Aims III, Onda). In accord with the simulations, the hydrophone scanned both the XZ and YZ planes, each within 10 mm $\times$ 40 mm in 0.15 mm steps. Compared to the simulations, which computed the resulting field element-wise, during the actual measurements, all transducers were excited at once to produce the total field.

### Determination of intensity

We registered the maximal pressure *P* over the time of the simulation at each location, and converted this value into intensity *I* using $I=\frac{P^2}{2 Z}$, where $Z=1.5$ MRayl is the acoustic impedance of water^43^. The intensity values were peak-normalized in all plots.

### Quantification of focal volume

We quantified the focal volume by measuring the total size of the intensity field above half the maximum value. Specifically, we took the convex hull of the voxels just exceeding the half-maximum intensity in both the XY and XZ planes. For each position on the x-axis, we calculated the full width half max—the width of the focal volume at half-maximum intensity—in the Y and Z dimension. We then integrated these products over the x axis to get the total volume. In particular, let the functions $FWHM_y(x)$ and $FWHM_z(x)$ denote the full width half max at position *x* in the Y and Z dimension, respectively. The focal volume then equals $\int ^{}_{} FWHM_y(x)FWHM_z(x) dx$.

## Data Availability

The data associated with the measurements are provided in the article. The data will be provided on request by emailing the corresponding author.

## Code availability

The code associated with the article is available and documented at onetarget.us/download/MFS.
